# A Sporozoite Asparagine-Rich Protein Controls Initiation of *Plasmodium* Liver Stage Development

**DOI:** 10.1371/journal.ppat.1000086

**Published:** 2008-06-13

**Authors:** Olivier Silvie, Kristin Goetz, Kai Matuschewski

**Affiliations:** Department of Parasitology, Heidelberg University School of Medicine, Heidelberg, Germany; Washington University School of Medicine, United States of America

## Abstract

*Plasmodium* sporozoites invade host hepatocytes and develop as liver stages (LS) before the onset of erythrocytic infection and malaria symptoms. LS are clinically silent, and constitute ideal targets for causal prophylactic drugs and vaccines. The molecular and cellular mechanisms underlying LS development remain poorly characterized. Here we describe a conserved *Plasmodium* asparagine-rich protein that is specifically expressed in sporozoites and liver stages. Gene disruption in *Plasmodium berghei* results in complete loss of sporozoite infectivity to rodents, due to early developmental arrest after invasion of hepatocytes. Mutant sporozoites productively invade host cells by forming a parasitophorous vacuole (PV), but subsequent remodelling of the membrane of the PV (PVM) is impaired as a consequence of dramatic down-regulation of genes encoding PVM-resident proteins. These early arrested mutants confer only limited protective immunity in immunized animals. Our results demonstrate the role of an asparagine-rich protein as a key regulator of *Plasmodium* sporozoite gene expression and LS development, and suggest a requirement of partial LS maturation to induce optimal protective immune responses against malaria pre-erythrocytic stages. These findings have important implications for the development of genetically attenuated parasites as a vaccine approach.

## Author Summary

*Plasmodium* parasites, the causative agents of malaria, have a complex life cycle that alternates between a mosquito vector and a vertebrate host. Infected mosquitoes transmit *Plasmodium* forms called sporozoites, which rapidly migrate to the host liver, invade hepatocytes, and differentiate into replicative liver stages (LS). After intensive multiplication, LS release merozoites that invade erythrocytes and cause malaria symptoms. *Plasmodium* LS are clinically silent, and represent ideal targets for prophylactic antimalarial drug and vaccine interventions. However, the molecular mechanisms underlying LS development remain poorly characterized. We describe here a *Plasmodium* protein, termed SLARP, which is specifically expressed in sporozoites and LS. In the absence of SLARP, sporozoites invade host cells normally but are then completely arrested at a very early stage of LS development. Our results indicate that SLARP functions as a specific regulator of the expression of genes involved in LS replication. Interestingly, early arrested *SLARP*-deficient parasites confer only limited protection in immunized mice, suggesting a requirement of parasite maturation to induce optimal protective immune responses against LS. Our study provides new insights into gene expression regulation during the complex life cycle of the malaria parasite and has important implications for the design of vaccines targeting *Plasmodium* liver stages.

## Introduction

With over 300 million cases each year, malaria remains the most important vector-borne infectious disease, severely affecting human health and social and economical development in endemic areas [1]. The malaria parasite *Plasmodium* is transmitted via the bite of a female *Anopheles* mosquito, which releases sporozoite stages into the skin [2]. Sporozoites enter the blood stream and, upon reaching the liver hepatocytes, transform into liver stages (LS), also called exo-erythrocytic forms (EEFs). LS grow, undergoing multiple rounds of nuclear divisions and ultimately produce thousands of first generation merozoites, which then commence the development of the pathogenic erythrocytic stages [3].

*Plasmodium* sporozoites invade hepatocytes by forming a membrane-bound specialized compartment, the parasitophorous vacuole (PV), where they differentiate into LS. LS are highly metabolically active, undergoing one of the fastest growth rates among eukaryotic cells. LS development is a complex process that includes initial sporozoite transformation, remodeling of the PV membrane (PVM), onset of mitotic divisions and parasite growth, before eventual merozoite formation and egress. LS constitute transition stages between sporozoites and merozoites, as reflected on transcriptome and proteome levels [4]. How the parasite regulates its gene expression to accomplish this critical transition phase remains elusive.

LS represent potential targets for causal prophylactic drugs and vaccines. In particular, immunization with radiation-attenuated parasites (RAPs) can induce sterile protection against sporozoite infection [5]. The recent demonstration that genetically attenuated parasites (GAPs) also confer protective immunity in mouse models created a renewed interest in whole parasite vaccine approaches against malaria [6],[7],[8]. Protective immunity induced by RAPs and GAPs relies primarily on CD8+ T cell responses against infected hepatocytes [9],[10],[11],[12],[13],[14],[15], but the antigenic specificity of protective CD8^+^ T cells is unknown.

Because of the high A/T nucleotide content of *Plasmodium* DNA, many malarial proteins contain low complexity regions (LCR). Interestingly, the composition of these LCR is biased towards an over-representation of asparagines as compared to lysines, although both share the same codon AT-richness, suggesting a phenotypic selection [16]. Still, the role of asparagine-rich proteins in *Plasmodium* remains unknown. Here we focused on a conserved *Plasmodium* asparagine-rich protein that is specifically expressed in sporozoites and early LS, and was therefore termed SLARP (Sporozoite and Liver stage Asparagine-Rich Protein). Parasites lacking *SLARP* develop normally in the mosquito and invade mammalian hepatocytes as efficiently as wild type parasites. Nevertheless, they are completely arrested at a very early step of LS development, prior to remodelling of the PVM and onset of nuclear divisions.

## Results

### *SLARP/S22* encodes a conserved asparagine-rich protein

**Figure 1:**
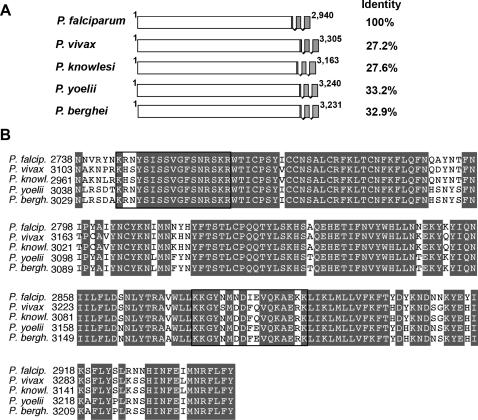
*SLARP* is conserved in *Plasmodium* species. (A) Genomic organization of representative *Plasmodium SLARP* genes. Shown are the *SLARP* gene loci for *P. falciparum* (Pf*SLARP*/PF11_0480), *P. vivax* (Pv*SLARP*/Pv092945), *P. knowlesi* (Pk*SLARP*/PKH_094440), *P. yoelii* (Py*SLARP*/PY03269, PY03923, Genbank accession no. EU579525) and *P. berghei* (Pb*SLARP*/PB000542.00.0, PB000547.01.0, Genbank accession no. EU579524). Sequence identity of the corresponding gene products are displayed on the right in comparison to *Pf*SLARP. (B) Alignment of the C-terminal end of *Plasmodium* SLARP proteins. Conserved residues are shaded in grey. The boxes indicate potential nuclear localization sequences (NLS) identified with PSORT II (http://psort.nibb.ac.jp).

*Plasmodium* liver stages (LS) are amongst the least known stages of the parasite life cycle. In an attempt to identify potential candidate genes specifically expressed in pre-erythrocytic stages, we previously used a suppressive subtractive hybridization screen in *P. yoelii*, which enabled the identification of 25 *S* genes expressed in sporozoites but not in blood stages [17]. One of these genes (*S22*) encodes an asparagine-rich protein that, as shown below, is specifically expressed in sporozoites and early/mid LS, and was therefore termed SLARP (Sporozoite and Liver stage Asparagine-Rich Protein). The *SLARP* gene and its genomic organization are conserved among *Plasmodium* species, including *P. falciparum* (Pf*SLARP*/PF11_0480), *P. vivax* (Pv*SLARP*/Pv092945), *P. knowlesi* (Pk*SLARP*/PKH_094440), *P. yoelii* (Py*SLARP*/Genbank accession no. EU579525) and *P. berghei* (Pb*SLARP*/Genbank accession no. EU579524). *SLARP* contains one large and two small exons, encoding a 2940–3305 amino acids protein with no apparent similarities to known proteins (**Fig. 1**). Remarkably, SLARP protein contains large stretches of low complexity, making up ∼40% of its sequence in *P. falciparum* and ∼30% in *P. berghei* and *P. yoelii*, with an overrepresentation of asparagine residues.

### SLARP is expressed in sporozoites and early/mid LS

**Figure 2:**
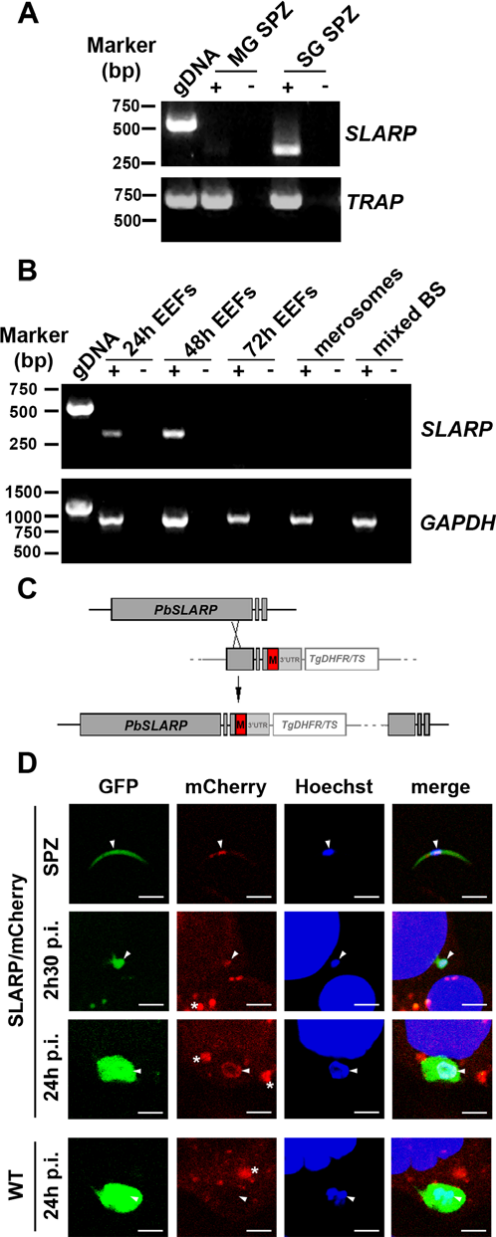
*SLARP* is expressed in *P. berghei* sporozoites and early/mid LS. (A) RT-PCR analysis of *SLARP* and *TRAP* expression in mosquito midgut (MG) and salivary gland (SG) sporozoites. gDNA, genomic DNA; +, with reverse transcription; −, without reverse transcription (B) RT-PCR analysis of *SLARP* and *GAPDH* expression in early (24 hours), mid (48 hours) and late (72 hours) LS, merosomes (recovered from the culture supernatants 72 hours post-infection), and mixed blood stages (BS). gDNA, genomic DNA; +, with reverse transcription; −, without reverse transcription. (C) Insertion strategy to generate the SLARP/mCherry parasites. The *PbSLARP* genomic locus was targeted with an integration plasmid containing a 3′ terminal fragment of the SLARP gene fused in frame to the mCherry coding sequence (M), the 3′ UTR of *PbDHFR/TS* (3′UTR) and the *TgDHFR/TS* selectable marker. Upon a single crossover event, the region of homology is duplicated, resulting in a functional, endogenous *PbSLARP* copy tagged with mCherry, followed by a truncated and non-expressed copy. (D) Detection of the SLARP/mCherry fusion protein (red) was analyzed directly by confocal fluorescence microscopy of sporozoites isolated from mosquito salivary glands (SPZ), or intracellular parasites 2h30 and 24 hours post-infection (p.i.) of HepG2 cells with SLARP/mCherry or WT *P. berghei* parasites constitutively expressing GFP (green). Nuclei were stained with Hoechst 33342 (blue). Bar = 5 µm. Note that the red fluorescent structures (indicated by asteriks) observed around SLARP/mCherry or WT parasites were also found in non-infected HepG2 cells, and correspond to host autofluorescent material unrelated to mCherry fluorescence (indicated by arrowheads), which was only observed inside SLARP/mCherry (but not WT) parasites.

According to *P. falciparum* DNA microarray data, Pf*SLARP* transcripts are readily detected in sporozoites but not in blood stages [18], consistent with the gene expression profiling in *P. yoelii* [17]. We investigated the expression of *SLARP* in different stages of the rodent parasite *P. berghei* using RT-PCR. *SLARP* transcripts were found in salivary gland sporozoites, as observed with *TRAP*, but were barely detectable in mosquito midgut oocysts (**Fig. 2A**). Sequencing of the RT-PCR products confirmed the predicted exon-intron gene structure (data not shown). *SLARP* expression was also detected in early (24h) and mid (48h) LS developing *in vitro* in HepG2 cells, but not in late LS (72h) (**Fig. 2B**). The final maturation of *P. berghei* LS *in vitro* is characterized by the appearance of detached infected cells, also called merosomes, in the culture supernatants [19]. *SLARP* expression was not detectable in merosomes, or in blood stages, whereas a control transcript (*P. berghei* glyceraldehyde 3-phosphate dehydrogenase, *GAPDH)* was detected throughout liver stage development (**Fig. 2B**).

In order to confirm *SLARP* expression timing and determine the subcellular localization of the protein, we generated a parasite line expressing the endogenous copy of *SLARP* fused in frame at its 3′ end to the coding sequence of the red fluorescent protein mCherry [20] (**Fig. 2C**). Transfection was performed in *P. berghei* ANKA parasites constitutively expressing GFP [21], leading to green fluorescent parasites that express a red fluorescent SLARP (PbSLARP/mCherry). Genotyping confirmed the desired integration event, and PCR on cDNA from PbSLARP/mCherry sporozoites showed expression and correct splicing of the fusion transcript (data not shown). We next assessed the expression of the fusion protein in different stages of PbSLARP/mCherry parasites by direct detection of mCherry fluorescence by confocal microscopy. As predicted, no red fluorescence was detectable in blood stages or oocysts of PbSLARP/mCherry parasites (data not shown). In contrast, mCherry could be detected in sporozoites and LS (**Fig. 2D**), in good agreement with the RT-PCR data. The mCherry signal in PbSLARP/mCherry parasites was rather weak, but was clearly distinguishable from background, and was not observed in WT parasites (**Fig. 2D**). Interestingly, mCherry fluorescence had a similar distribution as the Hoechst staining in PbSLARP/mCherry parasites (**Fig. 2D**). Although we could not determine precisely using confocal microscopy whether SLARP/mCherry localizes inside the nucleus or at its periphery, our data clearly indicate that the fusion protein associates with the parasite nucleus. Closer examination of the *Pb*SLARP primary structure revealed two potential bipartite nuclear localization signals (NLS) that are conserved in SLARP from other *Plasmodium* species and may contribute to the nuclear localization of the protein (**Fig. 1B**). Together our findings show that *Plasmodium* encodes a sporozoite and liver stage-specific asparagine-rich protein that seems to localize predominantly in the parasite nuclear region.

### *SLARP* gene deletion in *P. berghei*

**Figure 3:**
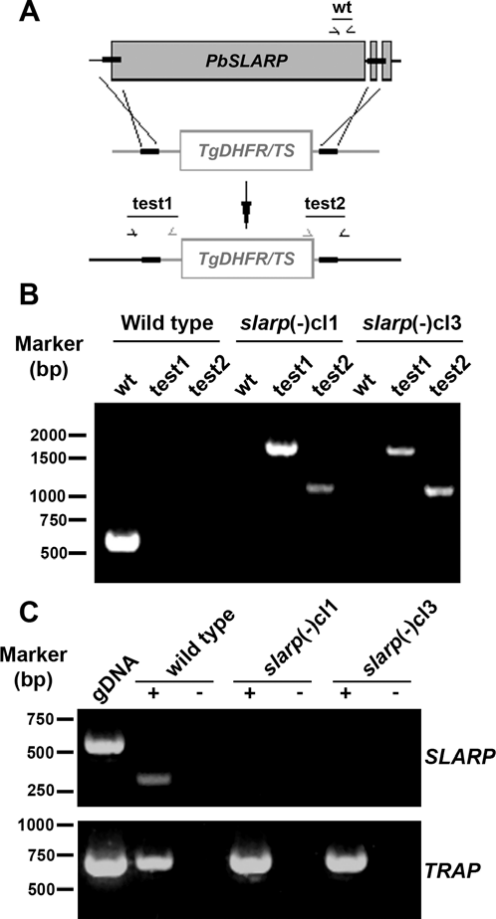
Targeted gene disruption of *P. berghei SLARP*. (A) Replacement strategy to generate the *slarp(-)* parasites. The wild-type (WT) *PbSLARP* genomic locus was targeted with a *Sac*II/*Kpn*I-linearized replacement plasmid containing 5’ and 3’ regions of the *SLARP* open reading frame (ORF) and the *Toxoplasma gondii dhfr/ts*-positive selectable marker. Upon a double crossover event, the *SLARP* gene is replaced by the selectable marker. Replacement- and wild type- specific test primer combinations and expected fragments (test1; test2; wt) are indicated. (B) Replacement-specific PCR analysis. Confirmation of the predicted gene targeting is achieved by specific primer combinations (test 1 and test 2), which can only amplify a signal from the recombinant locus. A wild type-specific PCR reaction confirms the absence of residual wild-type parasites in the clonal *slarp(-)* populations. (C) Depletion of *SLARP* transcripts in *slarp(-)* parasites. cDNA from wild-type and *slarp(-)* salivary gland sporozoites was amplified by PCR. Note the absence of a *SLARP*–specific signal in *slarp*(-) clones compared to a control transcript (*TRAP*), confirming the successful depletion of *SLARP*.

To study the importance of SLARP during the *Plasmodium* life cycle, we generated loss-of-function *P. berghei* parasites. We used a replacement strategy (**Fig. 3A**) to disrupt the endogenous *SLARP* gene copy by double crossover homologous recombination [22]. A targeting construct comprising 5′ and 3′ fragments of *SLARP* flanking the pyrimethamine-resistance cassette was used for positive selection after parasite transfection. The parental blood stage population from a successful transfection was used to isolate three independent disruptant clones, two of which were subsequently used for phenotypical analysis (*slarp*(-)cl1 and *slarp*(-)cl3). Occurrence of the double crossover was confirmed by PCR in pyrimethamine-resistant parasites, using primers specific for the 5′ and the 3′ end recombination events, respectively (**Fig. 3B**). The wild type locus was not detected in *slarp*(-)cl1 and *slarp*(-)cl3, confirming the homogeneity of the expected recombination. We also performed RT-PCR on RNA from salivary gland sporozoites of wild type and *slarp*(-) parasites. While *TRAP* control transcripts were detected in all parasite populations, *SLARP* transcripts were detected only in WT, but not in *slarp*(-) parasites, confirming the successful depletion of *SLARP* in the mutants (**Fig. 3C**).

### SLARP-deficient sporozoites are non-infective to rodents

We next examined the phenotype of *slarp*(-) parasites during the *P. berghei* life cycle. As expected from successful disruption of the *SLARP* gene in blood stages, *slarp*(-) clones were indistinguishable from WT parasites in development and growth of asexual stages (data not shown). *slarp*(-) parasites produced gametocytes and exflagellation of male gametocytes was similar to WT parasites (data not shown). Transmission to *Anopheles stephensi* mosquitoes and oocyst development were also normal when compared to WT parasites. *slarp*(-) oocysts produced sporozoites, which invaded mosquito salivary glands as efficiently as WT parasites (**Table 1**). Based on these findings we conclude that SLARP is dispensable for the *P. berghei* life cycle in the mosquito.

**Table 1:** *P. berghei slarp*(-) sporozoites invade mosquito salivary glands and mammalian hepatocytic cells.

Parasite population	Number of salivary gland sporozoites per mosquito^a^	Transmigration (% dextran-positive cells)^b^	Host cell invasion (Number of sporozoites)^c^
Wild type	10,486+/−1233	7.3+/−1.1	531+/−152
*slarp(-)* cl1	10,926+/−370	4.9+/−0.7	469+/−67
*slarp(-)* cl3	11,185+/−1153	5.7+/−0.7	453+/−100

a The mean number (+/−SD) of sporozoites was determined at day 19 after the infectious blood meal from at least three independent feeding experiments.

b The mean percentage (+/−SD) of dextran positive cells was determined by FACS analysis 3 hours after adding sporozoites to HepG2 cells.

c The mean number (+/−SD) of invaded sporozoites was determined by IFA 6 hours after addition of sporozoites to HepG2 cells.

We further investigated *slarp*(-) sporozoite *in vivo* infectivity to highly susceptible young Sprague-Dawley rats and C57BL/6 mice. In contrast to WT sporozoites, which consistently induced blood stage infection, none of the animals inoculated with *slarp*(-) sporozoites developed a patent blood stage infection, even after intravenous injection of very high numbers of sporozoites (up to 500,000), or when sporozoites were inoculated through mosquito bites, the natural transmission route (**Table 2**). These data demonstrate that disruption of *SLARP* in *P. berghei* abolishes sporozoite infectivity to rodents.

**Table 2:** *P. berghei slarp*(-) sporozoites are not infective to rodents.

Animals	Parasite population	Inoculum^a^	Number of infected animals	Prepatency (days)^b^
SD rats	Wild type	1,000	2/2	4
		10,000	2/2	3,5
		mosquito bites	2/2	4,5
	*slarp(-)* cl1	1,000	0/4	NA
		10,000	0/4	NA
		mosquito bites	0/4	NA
C57BL/6 mice	Wild type	1,000	2/2	4
		10,000	4/4	3
		100,000	4/4	3
		mosquito bites	3/3	3,3
	*slarp(-)* cl1	1,000	0/4	NA
		10,000	0/4	NA
		100,000	0/4	NA
	*slarp(-)* cl3	10,000	0/16	NA
		50,000	0/10	NA
		250,000	0/2	NA
		500,000	0/1	NA
		mosquito bites	0/5	NA

a Animals were injected intravenously with indicated numbers of sporozoites or exposed to the bites of 5–10 infected mosquitoes days 18–21 after the infectious blood meal.

b Number of days after sporozoite inoculation until detection of infected erythrocytes by microscopic blood smear examination.

NA: not applicable.

### Depletion of SLARP results in early developmental arrest after sporozoite invasion

**Figure 4:**
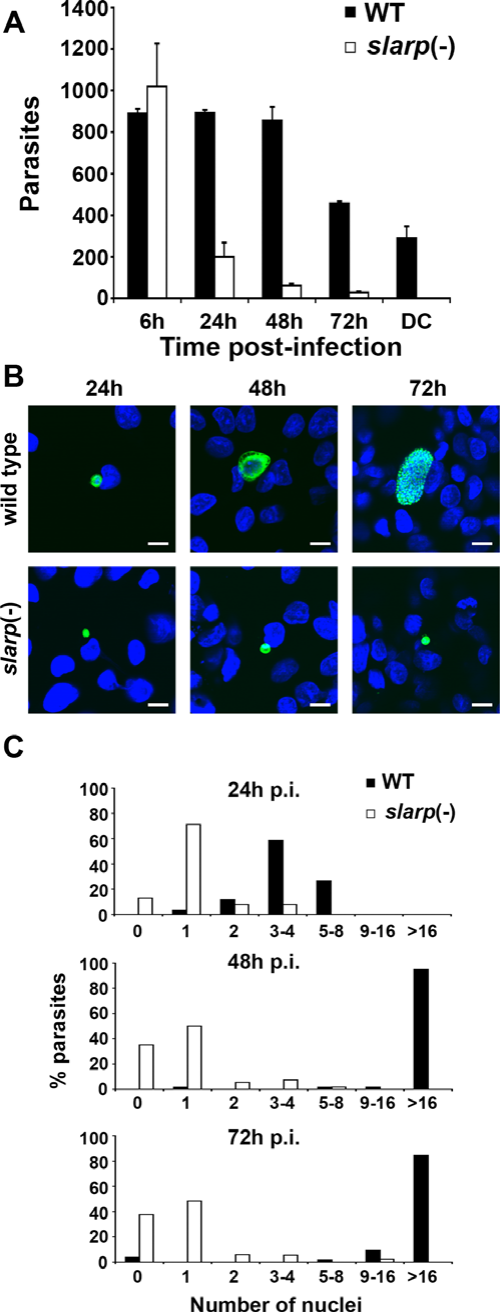
*slarp*(-) parasites are impaired in liver stage development. (A) HepG2 cells were infected with WT or *slarp*(-) sporozoites (1×10^4^) and cultured for 6–72 hours before staining with anti-CSP or anti-HSP70 antibodies and quantification by fluorescence microscopy. Results are expressed as the mean number of parasites from triplicate wells+/−SD. DC, detached infected cells. (B) HepG2 cells were infected with WT or *slarp(-)* sporozoites and cultured for 24, 48 or 72 hours before staining with anti-HSP70 antibodies (green) and Hoechst 33342 (blue), and examination by confocal fluorescence microscopy. Bar = 10 µm. (C) HepG2 cells were infected with WT or *slarp(-)* sporozoites and cultured for 24, 48 or 72 hours before staining with anti-HSP70 antibodies and Hoechst 33342. For each population, the number of nuclei in at least 50 parasites was determined by examination under a fluorescence microscope.

**Figure 5:**
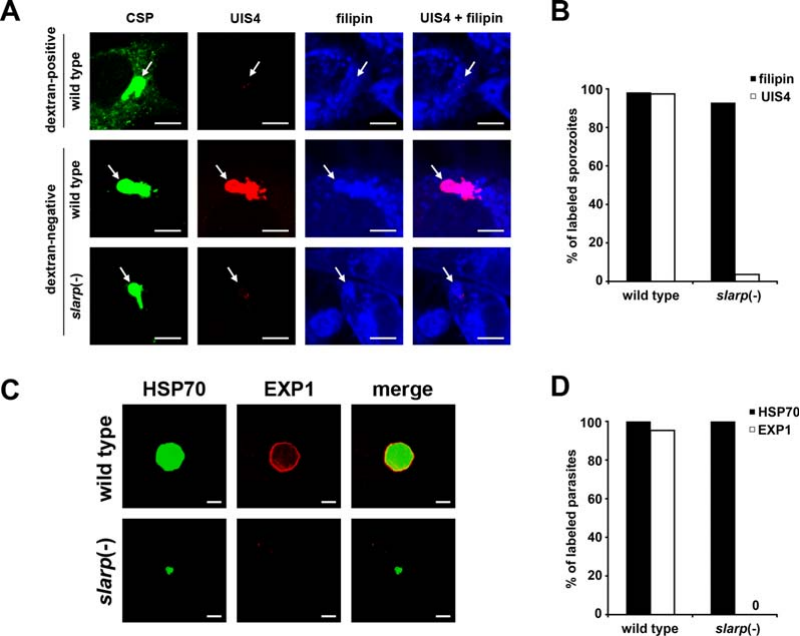
Remodelling of the PVM is impaired in *SLARP*-deficient parasites. (A) HepG2 cells were infected with WT or *slarp*(-) sporozoites in the presence of FITC-dextran and cultured for 6 hours before staining with anti-CSP (green), anti-UIS4 (red) and filipin (blue), followed by analysis by confocal fluorescence microscopy. Bar = 5 µm. Note that sporozoites inside dextran-positive cells were not stained by anti-UIS4 or filipin. (B) The percentage of UIS4 and filipin stained sporozoites was determined by examination of at least 50 dextran-negative infected cells for each population. (C) HepG2 cells were infected with WT or *slarp*(-) sporozoites and cultured for 48 hours before staining with anti-HSP70 (green) and anti-EXP1 (red) antibodies and analysis by confocal fluorescence microscopy. Bar = 10 µm. (D) The percentage of HSP70 and EXP1 labelled parasites was determined by examination of at least 50 GFP-positive 48h LS for each population.

Combined with the absence of a defect in blood stage multiplication, the lack of infectivity of *slarp*(-) sporozoites suggested a defect in sporozoite invasion and/or LS development. To distinguish which step was affected, we analyzed *slarp*(-) sporozoite infection *in vitro* in HepG2 cells. Sporozoites invade host cells by two alternative routes, cell traversal or productive infection [23]. Cell traversal is associated with host cell membrane rupture followed by sporozoite migration through the host cell cytoplasm, and is required for sporozoite migration in the skin and through the liver sinusoidal barrier [24],[25],[26],[27]. Productive infection of hepatocytes requires the formation of a PV, in which LS development takes place [23]. We analyzed these two different invasion modes *in vitro* with WT and *slarp*(-) sporozoites. As shown using a dextran-based wound and repair assay [23], cell traversal activity was not impaired in *slarp*(-) parasites, with similar numbers of dextran-positive cells as observed with WT (**Table 1**). The number of intracellular sporozoites early after infection was similar in *slarp*(-) clones and WT (**Fig. 4A** **and** **Table 1**), and most sporozoites of WT (81,9+/−0,4%, *n* = 204) and *slarp*(-) (81,5+/−0,8%, *n* = 211) were found in dextran-negative cells, providing indirect evidence that *slarp*(-) sporozoites are capable of productively entering the host cell. Staining with the cholesterol-binding agent filipin [28] confirmed the presence of a PVM in both WT and *slarp*(-) sporozoite-infected cells (see **Fig. 5A–B**).

The number of WT LS in the cultures was constant from 6 until 48 hours post-infection, followed by a marked decrease at 72 hours (**Fig. 4A**), which was concomitant with the appearance in the culture supernatants of detached infected cells (merosomes). Morphologically, WT parasite development was characterized by an increase in size over time and the occurrence of multiple nuclear divisions, as shown by Hoechst staining (**Fig. 4B and 4C**). At 72 hours post-infection, most WT parasites remaining in the culture monolayers displayed the typical aspect of mature segmented parasites (**Fig. 4B**). In contrast to WT parasites, the number of *slarp*(-) LS decreased over time *in vitro*, and we did not observe detachment of infected cells and release of merosomes (**Fig. 4A**). Furthermore, *slarp*(-) LS remained very small throughout the culture time, around 3–4 µm, which roughly corresponds to the size of 12–18h WT parasites (**Fig. 4B**). Most *slarp*(-) parasites were blocked at the one nucleus stage, even at late time points of infection (**Fig. 4C**). Notably, nuclear material was no longer detectable in a significant proportion of the mutant parasites, and this proportion increased over time (**Fig. 4C**). Taken together, these results demonstrate a severe defect of parasite replication in SLARP-deficient LS, which do not survive *in vitro*.

### Remodelling of the PVM is impaired in SLARP-deficient parasites

The early post-invasion defect of LS development and the PVM-positive filipin stain observed in *slarp*(-) parasites prompted us to analyze the expression of the up-regulated in infective sporozoites (UIS)-4 protein, which specifically localizes to the PVM early after invasion [6]. Invaded WT sporozoites displayed a strong UIS4 staining that extended to the host cell cytoplasm and colocalized with filipin (**Fig. 5A**), consistent with PVM localization. Importantly, no UIS4 or filipin staining of sporozoites was observed in infected dextran-positive cells (**Fig. 5A**). This confirms that UIS4 and filipin are specific markers of PVM formation, and further suggests that the bulk of UIS4 found in intracellular parasites is synthesized after sporozoite productive invasion. Remarkably, although *slarp*(-) sporozoites formed a PV inside host cells, as demonstrated by the absence of dextran uptake by the host cell and by staining of sporozoites by filipin, they lacked peripheral UIS4 staining (**Fig. 5A–B**). Instead, only weak internal UIS4 staining was sometimes observed in the mutant parasites, which likely corresponds to residual protein originating from sporozoites. We also analyzed expression of the Exported Protein-1 (EXP1), a mid/late LS antigen initially not present in sporozoites but expressed in LS, where, like UIS4, it localizes to the PVM [19],[29],[30]. EXP1 was detected by immunofluorescence in WT LS, with a circumferential staining consistent with PVM localization (**Fig. 5C**). As expected, EXP1 was not detected by immunofluorescence in salivary gland sporozoites or intracellular sporozoites 6 hours post-infection (data not shown). In contrast with WT parasites, we consistently failed to detect EXP1 in *slarp*(-) LS (**Fig. 5C–D**).

### Deletion of *SLARP* causes a dramatic decrease of *UIS3* and *UIS4* transcript abundance in sporozoites

**Figure 6:**
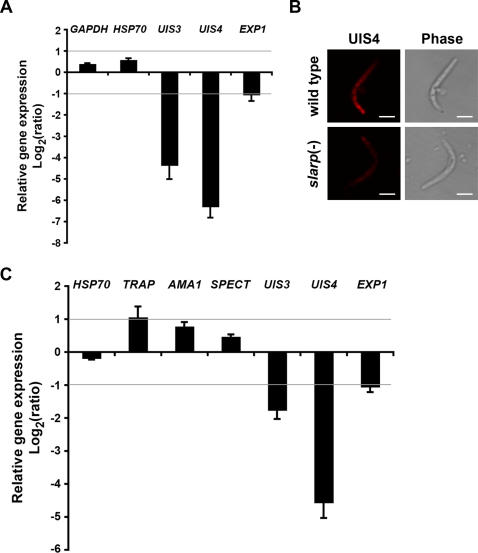
*UIS3* and *UIS4* gene expression is dramatically reduced in *SLARP*-deficient parasites. (A) Quantitative RT-PCR analysis of gene expression in infected HepG2 cell cultures, 6 hours post-infection with WT or *slarp*(-) sporozoites. Relative gene expression was normalized to the *GFP* expression level, and is expressed as the log2 of the ratio *slarp*(-)/WT (mean of three independent experiments+/−SD). (B) Air-dried WT and *slarp*(-) salivary gland sporozoites were stained with anti-UIS4 antibodies (red) before examination by confocal fluorescence microscopy, using the same settings as in Fig. 5A. Bar = 5 µm. (C) Quantitative RT-PCR analysis of gene expression in *slarp*(-) salivary gland sporozoites. Relative gene expression was normalized to the *GFP* expression level, and is expressed as the log2 of the ratio *slarp*(-)/WT (mean of three independent experiments+/−SD).

In order to determine whether the absence of UIS4 protein in invaded sporozoites was due to a defect in *UIS4* gene expression, we analyzed steady state transcript abundance in *slarp*(-) versus WT parasites, using quantitative PCR (RT-qPCR). We observed a dramatic reduction (∼80 fold) of *UIS4* transcript abundance in *slarp*(-) parasites as compared to WT parasites 6 hours post-infection, while control transcripts for *GAPDH* and heat shock protein 70 (*HSP70*) were not affected (**Fig. 6A**). RT-qPCR also demonstrated a ∼20 fold reduction in the level of transcripts for UIS3, another PVM-resident protein [7],[13], whereas *EXP1* expression was only slightly reduced (∼2 fold) (**Fig. 6A**).

Although *UIS3* and *UIS4* are required only during LS development, both are highly expressed already at the sporozoite stage [6],[7],[13],[31],[32]. UIS4 protein could be detected in *slarp*(-) sporozoites by immunofluorescence, but the labelling was consistently less intense than in WT sporozoites (**Fig. 6B**). Furthermore, RT-qPCR demonstrated a ∼20 fold decrease of *UIS4* transcript abundance in *slarp*(-) salivary gland sporozoites (**Fig. 6C**). As observed with invaded parasites, expression of *UIS3* and *EXP1* was also reduced in *slarp*(-) sporozoites collected from infected mosquito salivary glands, although to a lesser extent (∼3 fold and ∼2 fold, respectively) (**Fig. 6C**). Importantly, expression of several other genes was not significantly altered, including *HSP70* and genes involved in sporozoite invasion (*TRAP* and apical membrane antigen-1, *AMA1*) or transmigration (sporozoite protein essential for cell traversal, *SPECT*) (**Fig. 6C**). Altogether these results demonstrate that the lack of SLARP causes a selective and severe defect in expression of sporozoite genes involved in subsequent LS development.

### Immunization with SLARP-deficient sporozoites confers limited protective immunity against *P. berghei* infection in C57BL/6 mice

Several studies have demonstrated that immunization with GAPs can induce protective immune responses against sporozoite infection in mice [6],[7],[8],[13],[33]. Because genetic attenuation offers several advantages, notably in terms of reproducibility and stability of attenuation, GAPs constitute an interesting alternative for whole parasite vaccine approaches [34]. One essential requirement for a malaria GAP-based vaccine is the absence of breakthrough infections caused by the parasites used for immunization. Since *slarp*(-) sporozoites never cause blood stage infections (**Table 2**), they make interesting candidates as GAP vaccines. To test whether *SLARP*-deficient parasites are capable of inducing protective immunity, we immunized C57BL/6 mice with different doses of *slarp*(-) sporozoites, administered either by intravenous injection or through exposure to infective mosquito bites. Mice immunized with 50,000 *slarp*(-) sporozoites followed by two boost injections of 25,000 sporozoites were fully protected when challenged with WT parasites 6 weeks after the last boost (**Table 3**). However, protection rapidly decreased over time, since challenge 16 weeks after the last boost resulted in 40% protection only (**Table 3**). Reducing the dose of sporozoites used for immunization led to a progressive loss of protection. Sterile protection was only achieved in some of the mice immunized with 10,000 *slarp(-)* sporozoites, with a ∼2 days delay in patency in non-protected animals as compared to naïve mice, suggesting only partial elimination of WT parasites in the liver (**Table 3**). No protection was observed in the groups immunized with 1,000 *slarp*(-) sporozoites or by mosquito bites, where all mice developed a blood stage infection after challenge, with only a minor delay in patency as compared to naïve mice (**Table 3**).

**Table 3:** *P. berghei slarp*(-) sporozoites elicit limited protective responses in C57BL/6 mice against wild type sporozoite challenge.

Immunization doses^a^	Challenge dose (days after last boost)^b^	No. protected/No. challenged (%)	Prepatency (days)^c^
50,000/25,000/25,000	10,000 (d42)	5/5 (100%)	NA
50,000/25,000/25,000	10,000 (d98)	2/5 (40%)	(6,3)
10,000/10,000/10,000	10,000 (d23)	6/8 (75%)	(5)
10,000/10,000/10,000	10,000 (d36)	1/4 (25%)	(5,7)
1,000/1,000/1,000	10,000 (d36)	0/4 (0%)	4,25
Mosquito bites	Mosquito bites (d42)	0/5 (0%)	4,4
None	10,000	0/8 (0%)	3,4
None	Mosquito bites	0/3 (0%)	3,3

a C57BL/6 were immunized three times at two weeks intervals, with indicated numbers of *P. berghei slarp*(-) sporozoites injected intravenously, or by exposure to the bites of ten *slarp*(-) infected mosquitoes.

b Immunized mice were challenged by intravenous injection of 10,000 *P. berghei* WT sporozoites or by exposure to the bites of five WT infected mosquitoes, as indicated.

c Number of days after sporozoite inoculation until detection of infected erythrocytes by microscopic blood smear examination.

NA, not applicable.

## Discussion

Parasite replication in the liver is a prerequisite for the onset of malaria blood stage infection. Still, *Plasmodium* LS development remains poorly characterized on a molecular level. In this study, we identified a *Plasmodium* nuclear protein that is specifically expressed in sporozoites and LS and is vital for LS development. The developmental arrest observed in *slarp(-)* parasites is reminiscent of axenically cultured sporozoites that undergo initial transformation from elongated sporozoites to round early LS but fail to enter mitosis [35]. We hypothesize that this fundamental developmental switch is regulated, at least partially, by SLARP.

Our results demonstrate that the lack of SLARP causes a complete developmental arrest after sporozoite invasion. This phenotype is strikingly different from sporozoite gene knockouts reported so far [6],[7],[8],[26],[27],[36],[37],[38]. *Slarp*(-) sporozoites productively enter target cells under simultaneous formation of a replication-competent organelle, the PV. However, remodelling of the PVM is impaired as illustrated by the absence of two PVM-resident proteins, UIS4 and EXP1, in mutant parasites. It is unlikely that the defect in UIS4 expression alone is the cause for the developmental arrest observed in *SLARP*-deficient parasites. Indeed, LS development is not completely blocked in *UIS4*-knockout parasites, as evidenced by partial growth *in vitro* and occurrence of blood stage infections after sporozoite inoculation [6]. We rather hypothesize that defects in expression of multiple LS proteins (including UIS4, UIS3 and EXP1) account for the severe phenotype observed with *slarp*(-) parasites.

Regulation of gene expression in *Plasmodium* is poorly understood. Based on the paucity of specific transcription factors identified in *Plasmodium* genomes and on comparative analysis of transcriptome and proteome data, it was suggested that post-transcriptional mechanisms may play a major role in regulation of gene expression in *Plasmodium* [18],[39],[40],[41],[42]. In this regard, translation repression was recently documented in sexual stages of the *Plasmodium* life cycle [43], and may as well occur in other stages of the parasite life cycle. Nevertheless, the recent discovery of an Apetala-2 (AP2)-related family of transcription factors in *Plasmodium* raises the possibility that additional yet unidentified transcription regulators are also involved [44].

Several lines of evidence strongly support a role for SLARP in the regulation of gene expression in sporozoites and early LS. First, *SLARP* is expressed in sporozoites and early/mid LS, as shown by RT-PCR, and a tagged version of SLARP fused to the red fluorescent protein mCherry localizes predominantly to the parasite nucleus. Additional studies using specific antibodies will be required to determine whether this nuclear localization of SLARP/mCherry reflects the normal distribution of the endogenous SLARP, or represents an artefact resulting from the fusion to mCherry. We favor the former, since NLS are present in SLARP but not mCherry protein sequence, and various other parasite lines with different mCherry tagged proteins generated in our lab do not display nuclear localization of the fusion protein (E.D. Putrianti, O. Silvie and K. Matuschewski, unpublished data). Second, we demonstrate here using quantitative PCR that deletion of *SLARP* causes a dramatic decrease in steady state transcript abundance for at least two genes, *UIS3* and *UIS4*, in sporozoites collected from infected mosquito salivary glands or from HepG2 cells early after infection. This defect at the transcript level correlates with a dramatic reduction of the UIS4 protein content in invaded sporozoites, and to a lesser extent in salivary gland sporozoites, as shown by immunofluorescence.

Although *UIS3* and *UIS4* are essential only during LS development, they are highly expressed at the sporozoite stage [6],[7],[31],[32], suggesting that sporozoites in the mosquito salivary glands are already prepared for the next LS developmental step. Similarly to *UIS3* and *UIS4*, *SLARP* is expressed both in sporozoites and LS. While expression of *UIS3* and *UIS4* is dramatically diminished in *slarp*(-) parasites, expression of other sporozoites genes, involved in host cell traversal or productive invasion, is not affected, in good agreement with the absence of phenotypical defect during transmigration or host cell invasion by SLARP-deficient sporozoites. Based on these observations and the early developmental arrest of *slarp*(-) LS, we propose that SLARP functions as a specific regulator of the expression of genes involved at early steps of LS development, including *UIS3* and *UIS4* and probably additional yet unidentified genes.

The absence of EXP1 protein in *slarp*(-) LS probably involves a distinct mechanism, different from that responsible for *UIS4* (and *UIS3*) depletion. First, EXP1 is expressed in blood stages, whereas SLARP is not, demonstrating that SLARP is not required for EXP1 expression. Second, our data show that abundance of *EXP1* transcripts is only moderately affected in *slarp*(-) salivary gland as well as early invaded sporozoites. The fact that the protein EXP1 is not detected in sporozoites and very early LS (despite transcript presence) in WT parasites raises the possibility that EXP1 expression is regulated through post-transcriptional mechanisms. Since the lack of SLARP has only a modest effect on the expression of the *EXP1* gene, as evidenced by quantitative RT-PCR, we believe that the absence of EXP1 protein in *slarp*(-) parasites probably reflects an early blockage in the LS developmental program at a step that precedes the onset of *EXP1* translation activation. The lack of UIS4, which like EXP1 resides in the PVM, is probably not the cause of the defect in EXP1 expression in *slarp*(-) parasites since *uis4*(-) LS express EXP1 on their PVM normally [13].

So far, we cannot ascertain whether SLARP directly modulates *UIS3* and *UIS4* transcriptional level or is involved in upstream events that govern *UIS3* and *UIS4* gene expression. Although SLARP is characterized by a high content in asparagine residues, a feature shared by most *Plasmodium* AP2 putative transcription factors [44], as well as transcription regulators in other systems such as yeast [45], it does not contain any known conserved DNA binding domain. Whether SLARP acts as a specific transcription factor or regulates the activity of a specific transcription factor is still unknown. Alternatively, we cannot exclude that SLARP regulates sporozoite gene expression by other mechanisms, such as chromatin level regulation or post-transcriptional control.

Altogether our results demonstrate that SLARP is a key regulator of *Plasmodium* LS development, and plays a critical role in gene expression regulation at an early step of the infection process. The identification of an asparagine-rich protein as a master regulator of LS development raises the possibility that other uncharacterized LCR-containing proteins may play a similar role in other stages of the parasite life cycle. This study opens new perspectives for the elucidation of gene expression regulation during the complex life cycle of the malaria parasite and for the characterization of molecular mechanisms of parasite development inside host cells.

A key finding is the limited capacity of *slarp*(-) parasites to induce long-lasting sterilizing immune responses, irrespective of their ability to productively enter their final target cells. In particular, immunization with low numbers of *slarp*(-) sporozoites did not induce protection, in sharp contrast with *P36p*(-) parasites generated in the same strain, which under similar conditions were shown to fully protect immunized animals [46]. Although the poor protective efficacy of SLARP-deficient parasites as compared to other GAPs needs to be confirmed in side-by-side experiments, we speculate that this failure to induce strong protection is due to the lack of expression of antigens involved in protective immune responses. The nature of protective antigens against *Plasmodium* LS still remains elusive. T cell responses against the main sporozoite surface protein, the circumsporozoite protein (CSP), are believed to play an important role (reviewed in [47]). Nevertheless, two recent studies using transgenic mice or parasites clearly demonstrated that anti-CSP responses are dispensable for induction of sterile protection [48],[49], suggesting that additional antigens, most likely LS antigens, are required [50]. We could show here that in the absence of *SLARP*, LS development aborts before the expression of the EXP1 antigen, which has been identified as a protective antigen in *P. yoelii* [51]. We hypothesize that the early and complete developmental arrest observed in SLARP-deficient parasites causes a severe narrowing of their antigenic repertoire, and consequently of the immune responses they elicit. Similar to over-irradiated sporozoites, which are also inferior in eliciting protective immunity [52], *slarp(-)* parasites are perfectly attenuated. Although they represent ideal candidates for a safe GAP-based malaria vaccine high levels of protection may be very difficult to achieve. However, such a tool, an attenuated, yet poorly protective parasite, will be an important stepping stone towards the identification of protective liver stage antigens and immune effector cells. These studies can now be performed for the first time in side-by-side comparisons with fully protective GAPs generated in identical parasite strains [8],[46]. This path may ultimately lead to implementation of a safe metabolically active whole organism vaccine and new subunit strategies for antimalaria vaccine development.

## Materials and Methods

### Experimental animals, parasites and cell lines

Female NMRI mice, C57BL/6 mice and Sprague-Dawley (SD) rats were from Charles River Laboratories. All animal work was conducted in accordance with European regulations and approved by the state authorities (Regierungspräsidium Karlsruhe). We used *P. berghei* ANKA clone 507 parasites, which constitutively express the green fluorescent protein (GFP) [21]. HepG2 cells (ATCC HB-8065) were cultured as described [53].

### *Plasmodium berghei* transfection and genotypic analysis

For gene deletion, a 5′ and a 3′ fragment of *PbSLARP* were amplified by PCR from *P. berghei* genomic DNA, using primers *SLARP*rep1for (5′-TCCCCGCGGCTAACGCATATACCTATGATTCAGGACG-3′), *SLARP*rep2rev (5′-ATAAGAATGCGGCCGCGTTATGTATTTTTGTAAGAACTATTAAACC-3′), **SLARP*rep3for (5′-CCCAAGCTTCTTCACAAATATAATCAAACTTAGAACTGC-3′) and *SLARP*rep4for (5′-CGGGGTACCGAACTTCAAAATCATCCATATTATATCC-3′). B3D+ plasmid was obtained by inserting a 1214-bp fragment containing the 3′ untranslated region of *P. berghei dihydrofolate reductase/thymidylate synthase* (*DHFR/TS*) into *Bam*HI-*Eco*RI sites of the *P. berghei* targeting vector b3D.DT∧H.∧D (provided by Dr. Andrew Waters), which contains *Toxoplasma gondii DHFR/TS* as a selectable marker for resistance to pyrimethamine. Cloning of *P. berghei SLARP* 5′ and 3′ fragments into *Sac*II-*Not*I and *Hin*dIII-*Kpn*I sites, respectively, of B3D+ vector resulted into *SLARP*repB3D+ plasmid. Parasites were transfected with *Sac*II-*Kpn*I digested *SLARP*repB3D+ construct, using the Nucleofector® device (Amaxa GmbH) as described [21], injected intravenously into naïve NMRI mice, and selected by pyrimethamine treatment in the drinking water. Clonal parasite populations were obtained by limiting dilution series and intravenous injection of one parasite in 15 recipient NMRI mice. Genotyping of WT and *slarp*(-) parasites was performed by PCR on parasite genomic DNA using primers specific for the wt locus (*SLARP*wtfor, 5′-GAGACATATCAAATAATTACTACATACCACC-3′; *SLARP*wtrev, 5′-GGGGTTCATAATTATATTTTCATTAGGGTCC-3′), the 5’ (*SLARP*reptest1for, 5′-TCCCCGCGGCTAAAGTTCTCTATCGAATATAATATATACG-3′; UTRrev, 5′-AATTCCGGTGTGAAATACCGCACAGATGCG-3′) and the 3’ (TgPRO, 5′-CGCATTATATGAGTTCATTTTACACAATCC-3′; *SLARP*reptest2rev, 5′-CGGGGTACCCACACGAATGTGTATGCTTATGAAGATGG-3′) recombination events.*

### C-terminal fluorescent tagging

For fusion of the red fluorescent protein mCherry [20] to the C-terminus of SLARP, a 720-bp fragment corresponding to mCherry was first cloned into *SpeI*-*Bam*HI sites of B3D+ plasmid, resulting in B3D+mCherry vector. A PCR fragment corresponding to the last 2500-bp of *PbSLARP* was amplified with primers *SLARP*tagfor (5′-ATAAGAATGCGGCCGCTATGAGAAATACTGGGAATACCATTGAAGG-3′) and *SLARP*tagrev (5′-GGACTAGTATAAAATAAGAATCGATTCATTATTTCAAAATTTATATGG-3′), and cloned into *Not*I-*Spe*I sites of B3D+mCherry, resulting in the SLARPtagging construct. This targeting plasmid was linearized with *Hpa*I and transfected as described above. Naïve recipient mice were injected with transfected parasites and treated with pyrimethamine to select for recombinant PbSLARP/mCherry parasites. Integration of the construct in selected parasites was confirmed by PCR on genomic DNA using specific primer combinations. Expression of the SLARP/mCherry fusion protein was analyzed through direct detection of the red fluorescence of mCherry by confocal microscopy.

### Reverse transcriptase-PCR

Total RNA was purified from sporozoites, infected HepG2 cells or infected erythrocytes using the RNeasy kit (Qiagen). Reverse transcription was performed using the RETROscript kit (Ambion). cDNA was used as template for PCR amplification with primers specific for *P. berghei SLARP* (*SLARP*cDNAfor, 5′-TCCCCGCGGACAAGTTCAACAATCTTTTAATCACAGCGC-3′; *SLARP*cDNArev, 5′-ATAAGAATGCGGCCGCACACGAATGTGTATGCTTATGAAGATGGGG-3′), *TRAP* (*TRAP*cDNAfor, 5′-CCCGGATCCATGAAGCTCTTAGGAAATAG-3′; *TRAP*cDNArev, 5′-GTGTGGATCCTTCCTGACAAACTTTAGAAAG-3′), *UIS4* (*UIS4*cDNAfor, 5′-ACCCATTGATGAGACAAACGATTCAAAACC-3′; *UIS4*cDNArev, 5′-TCCATGTTATAAACGTTATTTCCTTCACCC-3′) or *GAPDH* (*GAPDH*cDNAfor, 5′-ATGGCAATAACAAAAGTCGGAATTAATGG-3′; *GAPDH*cDNArev, 5′-TGTGGATAGCCAAATCTAAAAGACGG-3′).

### Real time quantitative RT-PCR

Real time qPCR was performed on cDNA preparations using the ABI 7500 sequence detection system and Power SYBR® Green PCR Master Mix (Applied Biosystems), according to the manufacturer’s instructions. Three independent cDNA samples were tested for each population. qPCR was performed in triplicates, with 1 cycle of 95°C for 15 min, followed by 40 cycles of 95°C for 15 s, 55°C for 15 s, and 60°C for 45 s. Standard curves were generated for all primers using WT cDNA serial dilutions and gave amplification efficiencies of 90–100%. Data were analyzed with the SDS 1.3.1 software (Applied Biosystems). Relative transcript abundance was determined using the 2^−ΔΔCt^ method (User Bulletin 2, ABI). Expression data were normalized using the *GFP* gene. For analysis of *HSP70*, *UIS3*, *UIS4*, *TRAP*, *AMA1* and *SPECT* expression by RT-qPCR, we used the same primers as reported by Amino *et al*. [24]. Additional primers include: GFP forward, 5′-GATGGAAGCGTTCAACTAGCAGACC-3′; GFP reverse, 5′-AGCTGTTACAAACTCAAGAAGGACC-3′;GAPDH forward, 5′-AATTAAAGAAGCATCTGAGGGTCCAC-3′; GAPDH reverse, 5′-TTGAATATCCCCATTCATTGTCATACC-3′; EXP1 forward, 5′-AGGGAAGACATCCATTCCAAATTGG-3′; EXP1 reverse, 5′-TGAAGATTTGGCATGTTAAGTGGTG-3′.

### Sporozoite infectivity *in vivo*

SD rats or C57BL/6 mice were injected intravenously with 100 µl of sporozoite suspension of WT or *slarp*(-) parasites isolated from the salivary glands of infected *Anopheles stephensi* mosquitoes, or exposed to 5–10 infected mosquito bites, as indicated. Infection was then monitored daily by examination of Giemsa-stained blood smears. The delay of patency was defined as the time before detection of at least one erythrocytic stage in the smears.

### Phenotypic analysis of mutant parasites *in vitro*

For analysis of sporozoite cell traversal, HepG2 cells were incubated 3 hours at 37°C with sporozoites (5×10^4^) in the presence of 0.5 mg/ml fluorescein-conjugated dextran (Molecular Probes) [23]. Cells were then trypsinized and washed to remove extracellular sporozoites and dextran, and either analyzed by FACS to determine the percentage of dextran-positive cells, or plated in 8-chamber plastic Lab-Tek slides and further cultured at least 3 hours before analysis by fluorescence microscopy. To analyze productive infection and LS development, infected HepG2 cultures in Lab-Teks were incubated for 6–72 hours post-infection before analysis by immunofluorescence using primary antibodies against *P. berghei* CSP [54], HSP-70 [55], UIS4 [6], or EXP1 [19] as indicated, and appropriate secondary antibodies (Molecular probes). Hoechst 33342 (Molecular Probes) was used to stain nuclei. Cholesterol staining with filipin (Sigma) was performed as described [56]. Images were acquired on a Zeiss LSM510 confocal system (Zeiss, Germany) equipped with visible and UV laser lines, and processed with Adobe Photoshop software (Adobe Systems Inc.).

### Immunization and sporozoite challenge experiments

C57BL/6 mice were immunized three times at 2 weeks intervals with *slarp*(-) sporozoites injected intravenously. Animals were immunized by intravenous injection of 1,000, 10,000 or 50,000 sporozoites, followed by two boosts of 1,000, 10,000 or 25,000, respectively. For immunization through mosquito bites, mice were exposed to the bites of 10 infected mosquitoes, three times at 2 weeks intervals. Immunized mice were challenged at least 3 weeks after the last boost by intravenous injection of 10,000 *P. berghei* ANKA WT sporozoites or exposure to the bites of 5 infected mosquitoes. Infection was monitored daily by examination of Giemsa-stained blood smears until day 21 post-challenge. Non-immunized C57BL/6 mice were included in all challenge experiments to control WT sporozoite infectivity.

### Accession numbers

The nucleotide sequences reported in this paper have been submitted to the Genbank database with the accession numbers EU579524 and EU579525.
